# Measuring emotions in mathematics: the Achievement Emotions Questionnaire—Mathematics (AEQ-M)

**DOI:** 10.1007/s11858-022-01425-8

**Published:** 2022-10-26

**Authors:** Maik Bieleke, Thomas Goetz, Takuya Yanagida, Elouise Botes, Anne C. Frenzel, Reinhard Pekrun

**Affiliations:** 1 Department of Sport Science, University of Konstanz, 78457 Konstanz, Germany; 2 Department of Developmental and Educational Psychology, Faculty of Psychology, University of Vienna, Vienna, Austria; 3 Department of Psychology, Ludwig-Maximilians Universität München, Munich, Germany; 4 Department of Psychology, University of Essex, Colchester, UK; 5 Institute for Positive Psychology and Education, Australian Catholic University, Sydney, Australia

**Keywords:** Achievement Emotions Questionnaire Mathematics (AEQ-M), Control-value theory (CVT), Emotion components, Assessment

## Abstract

Understanding the structure, antecedents, and outcomes of students’ emotions has become a topic of major interest in research on mathematics education. Much of this work is based on the Achievement Emotions Questionnaire—Mathematics (AEQ-M), a self-report instrument assessing students’ mathematics-related emotions. The AEQ-M measures seven emotions (enjoyment, pride, anger, anxiety, shame, hopelessness, boredom) across class, learning, and test contexts (internal structure). Based on control-value theory, it is assumed that these emotions are evoked by control and value appraisals, and that they influence students’ motivation, learning strategies, and performance (external relations). Despite the popularity and frequent use of the AEQ-M, the research leading to its development has never been published, creating uncertainty about the validity of the proposed internal structure and external relations. We close this gap in Study 1 (*N* = 781 students, Grades 5–10, mean age 14.1 years, 53.5% female) by demonstrating that emotions are organized across contexts and linked to their proposed antecedents and outcomes. Study 2 (*N* = 699 students, Grade 7 and 9, mean age 14.0 years, 56.9% female) addresses another deficit in research on the AEQ-M, the lack of evidence regarding the assumption that emotions represent sets of interrelated affective, cognitive, motivational, and physiological/expressive components. We close this gap by evaluating extended AEQ-M scales, systematically assessing these components for five core mathematics emotions (enjoyment, anger, anxiety, hopelessness, boredom). Our work provides solid grounds for future research using the AEQ-M to assess emotions and their components in the domain of mathematics.

## The Achievement Emotions Questionnaire—Mathematics (AEQ-M)

Emotions play a pivotal role in academic settings and therefore constitute a major research topic in educational psychology (Pekrun & Linnenbrink-Garcia, 2014). Mathematics is an important domain of investigation in this regard (Schukajlow et al., 2017) for several reasons. Firstly, it is paramount to investigate students’ emotions regarding mathematics because it is a core subject and taught around the world. Secondly, mathematics is a domain to which students commonly attach rather high levels of perceived value (Goetz et al., 2014), which provides the basis for experiencing high levels of both negative (e.g., anxiety before a difficult test) and positive emotions (e.g., pride about receiving a good grade). Thirdly, the domain of mathematics is characterized by gender differences in various psychosocial variables, including the levels, antecedents, and outcomes of emotions (Frenzel et al., 2007a, 2007b; Goetz et al., 2013). Lastly, mathematics anxiety is an often researched topic (see, e.g., Ashcraft, 2002; Hembree, 1990). This established research tradition of examining mathematics anxiety might be fruitfully expanded by assessing other discrete emotions within the same learning context of mathematics.

However, there is still a lack of instruments assessing the core discrete emotions in the domain of mathematics. One exception is the Achievement Emotions Questionnaire—Mathematics (AEQ-M; Pekrun et al., 2005), a frequently used instrument that allows researchers to assess several emotions with a single instrument. The AEQ-M comprises a set of 60 self-report items, each presenting a statement about one of seven mathematics-related emotions (two positive emotions, namely, enjoyment and pride; five negative emotions, namely, anger, anxiety, shame, hopelessness, and boredom) and asking students to indicate the degree to which that statement applies to them personally. The items are organized in scales that cover emotions experienced during mathematics classes (e.g., “I enjoy my math class” is a sample item for class-related enjoyment), while learning for mathematics by oneself (e.g., “My math homework bores me to death” is a sample item for learning-related boredom), or while taking tests in mathematics (e.g., “When I have an upcoming math test, I get sick to my stomach” is a sample item for test-related anxiety). Students’ answers to the items pertaining to each emotion (e.g., ten items measuring enjoyment across class, learning, and test contexts) can be aggregated into composite scores and linked to various constructs of interest in research on mathematics education. For instance, Frenzel et al., (2007a, 2007b) showed that students’ AEQ-M scores for anxiety, anger, and shame were more strongly associated with academic achievement and parental expectations in China than in Germany, shedding light on important cultural differences.

Unlike instruments for assessing achievement emotions across school domains (e.g., the Achievement Emotions Questionnaire (AEQ); Pekrun et al., 2011), the AEQ-M and the data underlying its development have yet to be published. Instead, researchers have relied on a manual of the instrument that is available from its authors upon request (Pekrun et al., 2005). This is not a satisfactory state of affairs because it creates uncertainty about the validity of various assumptions made in research using the AEQ-M. The first assumption underlying the AEQ-M is that emotions can be organized into three different contexts, thus reflecting the internal structure of the AEQ-M. This organization pertains to the idea that emotions are context-dependent, that is, the experience of an emotion depends on whether students attend mathematics classes (class context), learn mathematics by themselves (learning context), or take tests in mathematics (test context). For instance, students might enjoy learning mathematics by themselves (i.e., high levels of learning-related enjoyment) more than attending mathematics classes and taking tests (i.e., low levels of class- and test-related enjoyment; Pekrun et al., 2002). Providing tentative support for this assumption about the internal structure of the AEQ-M, achievement emotions have been empirically shown to be organized within these contexts in research using the domain-general AEQ (Pekrun et al., 2011). However, the extent to which these findings can be transferred to the AEQ-M is an open question, casting doubt on whether mathematics-related emotions should be measured in a context-dependent way.

The second assumption is that achievement emotions are best understood as a set of interrelated affective, cognitive, motivational, and physiological/expressive processes that represent distinct components of the overall emotional experience (e.g., Scherer, 2009). Consequently, this assumption again pertains to the internal structure of the AEQ-M as it affects the content-domain of the items. For example, a comprehensive approach to measuring anxiety during a mathematics test might require items that ask students whether they feel anxious (affect), worry about their performance (cognition), want to escape the situation (motivation), and get queasy (physiological/expressive). The AEQ-M accounts for the assumed component structure of emotions in a non-systematic manner, tapping into different components of each emotion but failing to cover all components of all emotions. Thus, it has not been possible to date to investigate whether the component structure established for achievement emotions in general (Pekrun et al., 2011), may also pertain to the mathematics-related emotions measured with the AEQ-M.

The third assumption is grounded in control-value theory (CVT; Pekrun, 2006, 2018, 2021), which proposes that achievement emotions are linked to specific antecedents and outcomes. According to CVT, control and value appraisals are important antecedents of achievement emotions. Control appraisals pertain to students’ expectations of being able to initiate and perform achievement-related activities (e.g., studying for a mathematics test), expectations about whether these activities will produce desired outcomes (e.g., a good grade), and attributions regarding the controllability of the cause of outcomes that were attained (Pekrun, 2006, 2018). Appraisals of control are reflected in students’ academic self-concept (Shavelson et al., 1976) and self-efficacy (Bandura, 1977), two common measures of perceived control in empirical research (e.g., Goetz et al., 2012; Luo et al., 2016; for the relations between self-concept and self-efficacy in mathematics see Arens et al., 2022). In turn, value appraisals refer to the perceived value of academic activities and outcomes. Perceived value can relate to both extrinsic (e.g., importance of studying for attaining good grades) and intrinsic aspects of academic activities (e.g., interest in an activity). In addition, perceived value can also pertain to positive versus negative valence (e.g., importance of success vs. failure).

Regarding the outcomes associated with achievement emotions, these emotions are assumed to affect students’ learning and academic performance (Pekrun, 2006; Pekrun et al., 2011). Emotions can affect intrinsic and extrinsic motivation (e.g., learning out of curiosity versus learning to obtain good grades) and facilitate the use of flexible (e.g., elaboration of learning materials) and rigid learning strategies (e.g., rehearsal of materials). Moreover, emotions can affect the balance between students’ self-regulation (e.g., setting one’s own goals) and external regulation (e.g., seeking help from others). Importantly, these cognitive and motivational processes are assumed to mediate the effects of emotions on academic performance. Unlike its structural validity (i.e., context-dependency and component structure), the external relations of the AEQ-M along the lines of CVT have already been investigated in scattered studies using the instrument. For instance, there is evidence for control and value appraisals as interactive determinants of achievement emotions in mathematics (e.g., Putwain et al., 2018) and for the impact of mathematics-related achievement emotions on students’ learning and performance (e.g., Camacho-Morles et al., 2021). However, these relations have yet to be demonstrated with the data on which the development of the AEQ-M was based.

Therefore, the AEQ-M is based on several assumptions about the internal structure and external relations of mathematics-related emotions. It is difficult for readers to evaluate these assumptions, as neither the AEQ-M itself nor the data used for its development have been published thus far. Moreover, data permitting a systematic investigation of the proposed component structure of emotions are currently not available. This lack creates uncertainty about the psychometric properties and validity of the AEQ-M, and might impede the progress of research on the role of emotions in mathematics education. We aim to close these gaps by demonstrating the validity of the assumptions behind the AEQ-M in two studies, based on the data used for developing the AEQ-M (Study 1) and novel data with extended AEQ-M scales for enjoyment, anger, anxiety, boredom, and hopelessness (Study 2), through the following investigations. (1) We examine the assumed context-dependency of the emotions in Studies 1 and 2—that is, the assumption that discrete emotions (i.e., enjoyment, pride, anger, anxiety, shame, hopelessness, and boredom) differ between academic contexts (i.e., attending class, studying, and taking tests). (2) We introduce extended AEQ-M scales to examine the assumed component structure of emotions in Study 2—that is, the assumption that emotions represent sets of interrelated affective, cognitive, motivational, and physiological/expressive processes. These extended AEQ-M scales comprise 127 items measuring all four components of enjoyment, anger, and anxiety in class, learning, and test contexts, boredom in class and learning contexts, and hopelessness in test contexts. (3) We establish the external validity of the AEQ-M in Study 1 by investigating the relationship between emotions and their proposed core antecedents (control, value) and outcomes (motivation, learning strategies, achievement).

## Study 1

Study 1 is based on the data used for developing the AEQ-M. While scattered results from analyses of these data have been reported elsewhere (Goetz, 2004; Pekrun et al., 2005), a comprehensive and systematic analysis of the psychometric properties of the AEQ-M and its internal and external validity had not been conducted previously. To investigate the external validity of the AEQ-M, we followed the approach taken in the development of the domain-general AEQ (Pekrun et al., 2011) and assessed various measures of control and value appraisals (i.e., academic self-concept, self-efficacy, value of achievement, and interest), motivation (i.e., intrinsic motivation, achievement motivation, and effort), learning strategies (i.e., elaboration, rehearsal, self-regulation, and external regulation), and academic performance (i.e., grades).

### Methods

#### Sample

This study draws upon a sample of 781 German secondary school students (53.5% female, 46.5% male) from Grades 5 to 10 (Grade 5, *n* = 177; Grade 6, *n* = 103; Grade 7, *n* = 140; Grade 8, *n* = 149; Grade 9, *n* = 110; Grade 10, *n* = 102) with a mean age of *M* = 14.1 years (*SD* = 1.92). Students attended three different tracks referred to as *Hauptschule* (lowest track; *n* = 205 from 10 classrooms), *Realschule* (middle track; *n* = 270 from 10 classrooms), and *Gymnasium* (highest track; *n* = 306 from 12 classrooms).

#### Missing data

A total of 0.93% of data were missing, stemming from 279 incomplete records. The percentage of missing values across all variables ranged from 0.00 to 2.69%. Full information maximum likelihood (FIML) was used to deal with missing data (see Enders, 2010).

#### Measures

We used paper-and-pencil questionnaires with 5-point Likert Scales (1 = *not true at all*, 2 = *hardly true*, 3 = *somewhat true*, 4 = *largely true*, 5 = *exactly true*).

##### Achievement emotions

Achievement emotions were assessed with the Achievement Emotions Questionnaire—Mathematics (AEQ-M; Pekrun et al., 2005). It comprises 60 items (see Appendices 1 and 2) that measure seven achievement emotions in the domain of mathematics, namely, enjoyment, pride, anger, anxiety, shame, hopelessness, and boredom. Emotions are measured in terms of three contexts (class, learning, test), four components (affective, cognitive, motivational, physiological/expressive), and three points in time (before, during, after). However, not all contexts (e.g., test-related boredom) and components (e.g., affective test-related anger) are covered.

##### Antecedents of achievement emotions

Students’ academic self-concept, self-efficacy, performance-related valence, and interest were measured as antecedents of achievement emotions.

##### Academic self-concept

Three items measured students’ academic self-concept (e.g., “Mathematics is one of my best subjects”; Goetz, 2004; Marsh, 1990; α = 0.87).

##### Self-efficacy

Self-efficacy was measured with four items (e.g., “I am confident that I can master the skills taught in mathematics”; adapted from Kunter et al., 2002; α = 0.86).

##### Positive value of achievement

The positive value of achievement was measured with five items capturing the value of success (e.g., “It is very important for me to get a good grade in mathematics”; Goetz, 2004; α = 0.85).

##### Interest

Interest was assessed with eight items capturing the intrinsic value of activities (e.g., “Engaging in mathematics is one of my favorite activities”; Goetz, 2004; α = 0.90).

##### Outcomes of achievement emotions

We measured students’ motivation, learning strategies, and self-regulation and external regulation of learning as outcomes of achievement emotions.

##### Intrinsic and achievement motivation and effort regulation

Intrinsic and achievement motivation were assessed with three items (e.g., “In mathematics I do my homework because I like this subject”; α = 0.89) and two items (e.g., “I study for mathematics because I don't want to get bad grades”; Goetz, 2004; α = 0.75), respectively. Effort regulation was assessed with nine items (e.g., “I work hard to do well in mathematics classes even if I do not like what we are doing”; Wild & Schiefele, 1994; α = 0.79).

##### Learning strategies

Elaboration and rehearsal were measured with nine items (e.g., “When I study for mathematics, I try to connect the material to things I've already learned in other subjects”; α = 0.86) and four items (e.g., “When I study for mathematics, I practice by reciting formulas over and over”; adapted from Baumert et al., 1997; Kunter et al., 2002; α = 0.75), respectively.

##### Self- and other-regulated learning

Self-regulated and externally regulated learning was assessed with nine items (e.g., “When studying for mathematics, I set my own goals that I want to achieve”; modified from Goetz, 2004; α = 0.83) and six items (e.g., “In the way I solve my mathematics problems, I follow my teacher's recommendations exactly”; modified from Goetz, 2004; α = 0.74), respectively.

##### Academic achievement

Students reported their last midterm mathematics grade. Grades ranged from 1 (*very good*) to 6 (*insufficient*) and were inverted for ease of interpretation, so that higher values corresponded to better achievement.

#### Analytic strategy

**Fig. 1:**
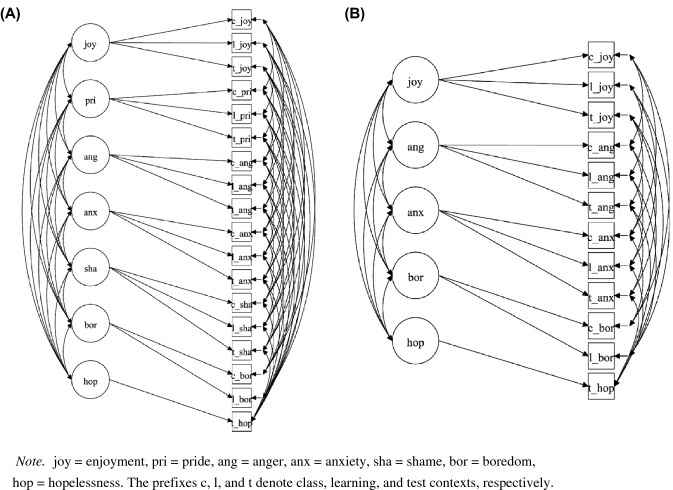
Model 4 with seven factors (**A**; Study 1) and five factors (**B**; Study 2)

A series of confirmatory factor analyses (Brown, 2015) was conducted to investigate the structural relationships between emotions. First, a total of four CFA models representing different hypotheses about these relationships were estimated (analogous to Pekrun et al., 2011), as follows: one general bipolar factor across all contexts and emotions (M1); seven factors representing each emotion (M2); three factors representing each context (M3); and seven factors representing each emotion and correlated uniqueness within settings (M4; see Fig. 1). Second, we computed latent correlations of emotions with control and value appraisals, motivation, strategies, and performance based on single indicator models with model-based corrections for unreliability (see Cole & Preacher, 2014). Measurement models were evaluated using the fit indices CFI, TLI, RMSEA, and SRMR based on common cut-off criteria (CFI and TLI ≥ 0.95, SRMR ≤ 0.08, RMSEA ≤ 0.05; see Kline, 2016). In addition, the Bayesian information criterion (BIC) was used to select among competing models, where a lower BIC value indicates a better trade-off between model fit and model complexity.

Models were estimated with Mplus 8.4 (Muthén & Muthén, 1998–2017) using the robust maximum likelihood estimation method (MLR) with chi-square test statistic and standard errors taking into account non-independence of observations due to students nested in classrooms.

### Results and discussion

We observed higher means for positive than for negative emotions, sufficient variation in item scores, and low levels of skewness and kurtosis (Table 1). All scales displayed good or very good reliability, 0.84 ≤ α ≤ 0.91. The positive emotions of enjoyment and pride were positively correlated, *r* = 0.78, and the negative emotions of anger, anxiety, shame, hopelessness, and boredom were also positively correlated, 0.25 ≤ *r* ≤ 0.86. Positive and negative emotions were negatively correlated, − 0.62 ≤ *r* ≤  − 0.14.

**Table 1:** Descriptive statistics and zero-order correlations of the AEQ-M scales

Scale	Items	*M*	*SD*	Skew	Kurt	$\overline{r}_{i(t - i)}$	α	Correlations					
								1	2	3	4	5	6
1 Enjoyment	10	2.76	0.88	0.32	− 0.45	0.64	0.90						
2 Pride	6	2.77	1.02	0.27	− 0.63	0.67	0.87	0.78					
3 Anger	9	2.45	0.97	0.51	− 0.50	0.62	0.88	− 0.62	− 0.43				
4 Anxiety	15	2.29	0.85	0.63	− 0.15	0.61	0.91	− 0.45	− 0.34	0.70			
5 Shame	8	1.90	0.80	1.09	0.92	0.57	0.84	− 0.26	− 0.14	0.52	0.72		
6 Hopelessness	6	2.19	1.04	0.83	− 0.11	0.70	0.89	− 0.50	− 0.41	0.70	0.86	0.68	
7 Boredom	6	2.50	1.11	0.56	− 0.58	0.72	0.89	− 0.59	− 0.39	0.69	0.35	0.25	0.40

$\overline{r}_{i(t - i)}$ denotes the average part-whole corrected item-total correlation. All correlation coefficients were statistically significant at α = 0.05

#### Structural relationships

In order to examine structural relationships between emotions, four CFA models were estimated (see Table 2). Results showed that the model representing the two-facet structure of the instrument (i.e., seven emotions nested within three contexts; M4) showed an acceptable model fit (χ^2^(70) = 244.78, CFI = 0.978, TLI = 0.951, RMSEA = 0.057, SRMR = 0.041) according to all fit indices, as well as the smallest BIC. This result indicates the best trade-off between model fit and model complexity among the four competing models. This finding is in line with CVT, showing that several discrete achievement emotions can be distinguished and that they are context-dependent.

**Table 2:** Confirmatory factor analysis: model comparison

Model	${\chi }^{2}$	df	CFI	TLI	RMSEA	SRMR	BIC
M1: One-Emotion Factor Model	3736.49	135	0.541	0.480	0.185	0.138	35582.28
M2: Seven-Emotion Factor Model	992.08	115	0.888	0.851	0.099	0.058	32756.47
M3: Three-Context Factor Model	3464.42	132	0.575	0.508	0.180	0.144	35255.77
M4: Seven-Emotion x Three-Context Factor Model	**244.78**	**70**	**0.978**	**0.951**	**0.057**	**0.041**	**32135.69**

*N* = 781. Seven emotions are enjoyment, pride, anger, anxiety, shame, hopelessness, and boredom. Three contexts are class, learning, and test. Model selected by the BIC is shown in boldface.

#### Correlations with external criteria

As expected and in line with CVT (Pekrun, 2006), enjoyment and pride were positively associated with all external criteria (Table 3), indicating that higher levels of positive emotions are related to higher levels of control and value appraisals, higher levels of motivation, more frequent use of learning strategies, and better academic performance. Anger, anxiety, shame, hopelessness, and boredom, on the other hand, were in general negatively associated with control and value appraisals, motivation, and performance. These results mirror research with the domain-general AEQ (Pekrun et al., 2011) and the AEQ-M (e.g., Frenzel et al., 2007a, 2007b; Putwain et al., 2018).

**Table 3:** Latent correlations of emotions with appraisals, motivation, strategies, and performance

Emotion	Control and value appraisals					Motivation				Learning strategies					Performance
	Academic self-concept	Self-efficacy	Positive value of achievement	Interest		Intrinsic motivation	Achievement motivation	Effort		Elaboration	Rehearsal	Self-regulation	External regulation		Grade
Enjoyment	**0.73**	**0.70**	**0.46**	**0.91**		**0.93**	**0.40**	**0.62**		**0.63**	**0.26**	**0.71**	**0.49**		**0.46**
Pride	**0.67**	**0.66**	**0.44**	**0.80**		**0.78**	**0.40**	**0.57**		**0.62**	**0.30**	**0.69**	**0.49**		**0.43**
Anger	− **0.56**	**− 0.56**	**− 0.27**	**− 0.57**		**− 0.60**	**− 0.25**	**− 0.39**		**− 0.33**	0.04	**− 0.43**	**− 0.16**		**− 0.38**
Anxiety	**− 0.60**	**− 0.60**	0.05	**− 0.34**		**− 0.39**	− 0.05	**− 0.11**		**− 0.12**	**0.27**	**− 0.26**	**0.11**		**− 0.45**
Shame	**− 0.41**	**− 0.45**	0.05	**− 0.15**		**− 0.22**	− 0.05	**− 0.12**		− 0.02	**0.28**	**− 0.21**	**0.16**		**− 0.35**
Hopelessness	**− 0.65**	**− 0.64**	− 0.09	**− 0.36**		**− 0.43**	**− 0.17**	**− 0.25**		**− 0.18**	**0.19**	**− 0.33**	0.01		**− 0.49**
Boredom	**− 0.37**	**− 0.36**	**− 0.40**	**− 0.61**		**− 0.64**	**− 0.35**	**− 0.56**		**− 0.44**	**− 0.21**	**− 0.42**	**− 0.32**		**− 0.17**

*N* = 781. Higher numbers in grade indicate better performance; statistically significant coefficient at α = 0.05 are shown in boldface

However, there are noteworthy exceptions from this general pattern. Anxiety, shame, and hopelessness correlated positively with declarative repetition, and anxiety and shame correlated positively with external regulation of learning. These negative emotions might prompt students to use more rigid study strategies and to seek help in order to prevent failure. Anxiety may stimulate learning and performance by promoting extrinsic motivation (e.g., Bieleke et al., 2022), but hamper self-regulation and performance by overtaxing cognitive resources (e.g., through processing worry cognitions; Roos et al., 2021a, 2021b), resulting in variable associations with performance (Pekrun, 2018). Interestingly, anger and boredom were negatively associated with perceived positive value of achievement, whereas anxiety, shame, and hopelessness did not relate to positive achievement value. The negative link between value and boredom is in line with CVT propositions (i.e., boredom is generally linked to low levels of value; Pekrun, 2006).

## Study 2

In Study 2, we developed extended scales for enjoyment, anger, anxiety, hopelessness, and boredom as essential emotions in mathematics, systematically covering all four components (i.e., affective, cognitive, motivational, and physiological/expressive). This allowed us to establish the overall structural validity of the AEQ-M by examining the robustness of the confirmatory factor analyses conducted in Study 1. More importantly, we could investigate the structural validity of each scale. In line with research on the domain-general AEQ (Pekrun et al., 2011), we expected that models with four correlated components (i.e., four-component models) and models with four second-order components governed by a higher-order factor representing the emotion (i.e., hierarchical models) fit the data better than models with a single factor representing the emotion (i.e., single-factor models). The former two models represent the idea that emotions are sets of interrelated affective, cognitive, motivational, and physiological/expressive components, one of the assumptions underlying the AEQ-M (e.g., Lange & Zickfeld, 2021; Scherer, 2009). The latter model represents the idea that emotions are unitary constructs with no distinguishable components.

### Methods

#### Sample

This study draws upon a sample of 699 German secondary school students (56.9% female, 41.1% male) from Grade 7 (*n* = 83) and Grade 9 (*n* = 616) with a mean age of *M* = 14.0 years (*SD* = 0.9). Students attended three different tracks referred to as *Hauptschule* (lowest track; *n* = 205 in Grade 9), *Realschule* (middle track; *n* = 83 in Grade 7, *n* = 203 in Grade 9), and *Gymnasium* (highest track; *n* = 208 in Grade 9).

#### Measures

The construction of items for the extended AEQ-M scales was based on the same qualitative interviews and pilot studies that were used to construct the AEQ-M (Goetz, 2004; Molfenter, 1999; Titz, 2001). We used paper-and-pencil questionnaires with 5-point Likert Scales (1 = *not true at all*, 2 = *hardly true*, 3 = *somewhat true*, 4 = *largely true*, 5 = *exactly true*).

#### Missing data

A total of 0.27% of data were missing, stemming from 141 incomplete records. The percentage of missing values across all variables ranged from 0.00% to 1.14%. Full information maximum likelihood (FIML) was used to deal with missing data (see Enders, 2010).

##### Achievement emotions

Enjoyment, anger, anxiety, boredom, and hopelessness were assessed with 125 items (Appendix 2). These extended AEQ-M scales supplemented the existing scales by additional items to represent all emotion components (e.g., the affective, cognitive, motivational, and physiological/expressive components of class-related boredom). As in the AEQ-M, boredom was measured only in class and learning contexts, and hopelessness was measured only in test contexts.

#### Analytic strategy

Firstly, we analyzed the same set of four CFA models as in Study 1. Secondly, the component structure was investigated for each of the five emotions and three contexts by estimating three CFA models for each combination of emotion and context: A model with one general factor across all components (M1), a model with four factors representing each component (M2), and a second-order factor model based on the four factors representing each component (M3). This two-step approach facilitates comparisons between Studies 1 and 2 as well as with previous research on the validation of the domain-general AEQ (e.g., Bieleke et al., 2021; Pekrun et al., 2011), which involved an analogous approach to examine the structural validity of the AEQ.

### Results and discussion

As in Study 1, we observed higher levels of positive than negative emotions, sufficient variation in item scores, and low levels of skewness and kurtosis (Table 4). All scales displayed good to very good reliability, 0.91 ≤ α ≤ 0.96. The negative emotions of anger, anxiety, hopelessness, and boredom were positively correlated, 0.31 ≤ *r* ≤ 0.84, and negatively correlated with enjoyment, − 0.61 ≤ *r* ≤  − 0.26.

**Table 4:** Descriptive statistics and zero-order correlations of the AEQ-M scales

Scale	Items	*M*	*SD*	Skew	Kurtosis	$\overline{r}_{i(t - i)}$	α	Correlations			
								1	2	3	4
1. Enjoyment	30	2.44	0.67	0.47	0.04	0.57	0.94				
2. Anger	31	2.24	0.80	0.75	0.09	0.62	0.95	− 0.47			
3. Anxiety	33	2.15	0.72	0.76	0.23	0.60	0.95	− 0.26	0.65		
4. Hopelessness	22	2.08	0.89	0.85	0.02	0.68	0.91	− 0.42	0.70	0.84	
5. Boredom	9	2.82	0.99	0.34	− 0.75	0.72	0.96	− 0.61	0.69	0.31	0.46

$\overline{r}_{i(t - i)}$ denotes the average part-whole corrected item-total correlation. Items were answered on 5-point Likert Scale (1 = *not true at all,* 5 = *exactly true*). All correlation coefficients were statistically significant at α = 0.05

#### Structural relationships

Among the four CFA models we compared (Table 5), the model representing the two-facet structure of the instrument (i.e., five emotions nested within three contexts, M4) again provided the best fit to our data, and the best trade-off between model fit and complexity among the four competing models. The model fit according to CFI, TLI, and SRMR was acceptable, whereas the RMSEA exceeded the threshold for acceptable model fit (χ^2^(27) = 213.04, CFI = 0.969, TLI = 0.925, RMSEA = 0.099, SRMR = 0.046).

**Table 5:** Confirmatory factor analysis: model comparison

Model	${\chi }^{2}$	df	CFI	TLI	RMSEA	SRMR	BIC
M1: One-Emotion Factor Model	2779.00	54	0.552	0.452	0.269	0.146	17110.11
M2: Five-Emotion Factor Model	808.43	45	0.874	0.816	0.156	0.056	15040.46
M3: Three-Context Factor Model	2761.59	51	0.554	0.423	0.276	0.148	16984.23
**M4: Five-Emotion x Three-Context Factor Model**	**213.04**	**27**	**0.969**	**0.925**	**0.099**	**0.046**	**14491.45**

*N* = 699. Five emotions are enjoyment, anger, anxiety, hopelessness, and boredom. Three contexts are class, learning, and test. Model selected by the BIC is shown in boldface

#### Component structure

As expected, the component factor and the hierarchical models fit our data well (Table 6) and were superior to single-factor models in all cases except learning-related anger and boredom. In these latter two cases, however, the fit of the component factor and the hierarchical models were also very good. In general, the best fitting models provided acceptable fit to the data in absolute terms with only few exceptions (e.g., enjoyment). These findings suggest that emotions measured with the extended AEQ-M scales capture the component structure predicted by the control-value theory.

**Table 6:** Emotion component structure of AEQ scales: confirmatory factor analysis

Emotion	Model	Class-related emotions							Learning-related emotions							Test-related emotions						
		${\chi }^{2}$	df	CFI	TLI	RMSEA	SRMR	BIC	${\chi }^{2}$	df	CFI	TLI	RMSEA	SRMR	BIC	${\chi }^{2}$	df	CFI	TLI	RMSEA	SRMR	BIC
Enjoyment	M1	169.63	35	0.944	0.928	0.074	0.042	17237.58	413.24	35	0.792	0.733	0.124	0.086	19115.42	455.91	35	0.818	0.767	0.131	0.082	19575.14
	M2	89.57	29	0.975	0.961	0.055	0.031	17167.59	**197.49**	**29**	**0.907**	**0.856**	**0.091**	**0.054**	**18848.05**	**219.42**	**29**	**0.918**	**0.873**	**0.097**	**0.058**	**19355.64**
	M3	**92.72**	**31**	**0.974**	**0.963**	**0.053**	**0.031**	**17163.05**	240.93	31	0.885	0.833	0.098	0.066	18902.21	247.44	31	0.907	0.864	0.100	0.065	19382.49
Anger	M1	121.79	44	0.966	0.957	0.050	0.030	20295.23	**120.59**	**36**	**0.960**	**0.951**	**0.058**	**0.035**	**21190.63**	154.89	35	0.944	0.928	0.070	0.039	19452.87
	M2	91.28	38	0.977	0.966	0.045	0.027	20288.66	103.68	29	0.965	0.946	0.061	0.031	21213.07	**73.90**	**29**	**0.979**	**0.967**	**0.047**	**0.025**	**19382.14**
	M3	**93.22**	**40**	**0.977**	**0.968**	**0.044**	**0.028**	**20283.83**	99.00	31	0.968	0.954	0.056	0.031	21203.55	90.80	31	0.972	0.959	0.053	0.030	19396.28
Anxiety	M1	344.87	54	0.879	0.852	0.088	0.054	20727.80	308.45	44	0.863	0.829	0.093	0.059	19992.19	203.40	35	0.937	0.919	0.083	0.039	20936.47
	M2	**212.82**	**48**	**0.931**	**0.906**	**0.070**	**0.044**	**20572.89**	**175.49**	**38**	**0.929**	**0.897**	**0.072**	**0.044**	**19806.99**	**49.07**	**29**	**0.992**	**0.988**	**0.031**	**0.018**	**20782.33**
	M3	247.87	50	0.918	0.891	0.075	0.050	20622.95	203.14	40	0.916	0.884	0.076	0.050	19852.66	64.86	31	0.987	0.982	0.040	0.023	20788.29
Hopelessness	M1															95.44	27	0.968	0.957	0.060	0.028	16664.80
	M2															65.98	21	0.979	0.964	0.055	0.024	16661.98
	M3															**69.20**	**23**	**0.978**	**0.966**	**0.054**	**0.025**	**16654.23**
Boredom	M1	251.61	54	0.953	0.943	0.072	0.032	22378.91	**94.60**	**35**	**0.980**	**0.974**	**0.049**	**0.022**	**20108.36**							
	M2	**215.21**	**48**	**0.961**	**0.946**	**0.071**	**0.031**	**22368.67**	76.85	29	0.984	0.975	0.049	0.020	20121.73							
	M3	228.10	50	0.958	0.945	0.071	0.031	22371.55	82.52	31	0.983	0.975	0.049	0.021	20120.89							

*N* = 699. Models selected by the BIC are shown in boldface

## Discussion

The Achievement Emotions Questionnaire—Mathematics (AEQ-M) is an important instrument for assessing a broad range of emotions in mathematics. Despite its popularity and frequent use in research on mathematics education, however, the instrument has yet to be published and evidence for several underlying assumptions is either missing or scattered across the literature. Specifically, there is a dearth of evidence for the context-dependence of mathematics-related emotions (i.e., emotions differ between class, learning, and test contexts), their component structure (i.e., emotions reflect a set of interrelated affective, cognitive, motivational, and physiological/expressive processes), and their associations with antecedents (control, value) and outcomes (motivation, learning strategies, achievement) assumed by the control-value theory of achievement emotions (CVT; Pekrun, 2006). In the present research, we capitalized on both the data originally used to develop the AEQ-M (Study 1) and additional data (Study 2) to scrutinize the validity of these assumptions. Regarding the structural validity of the AEQ-M (i.e., context-dependency, component structure), both studies provided evidence that mathematics-related emotions assessed with the AEQ-M are indeed context-specific. As such, emotions measured in one context might differ from emotions measured in another context (e.g., students might experience more anxiety in tests than in classes). Moreover, Study 2 suggests that mathematics-related emotions are best understood as reflecting a set of interrelated processes. For instance, experiencing anxiety means that students feel anxious (affective), are worried (cognitive), want to leave (motivational), and get queasy (physiological/expressive). These findings corroborate and extend previous research that investigated some of these assumptions about the internal structure of mathematics-related emotions (e.g., context-dependency in a Portuguese version of the AEQ-M; Moreira et al., 2019). Moreover, they provide a novel set of extended AEQ-M scales for assessing enjoyment, anger, anxiety, hopelessness, and boredom in mathematics education research.

Regarding the external validity of the AEQ-M, the results of Study 1 showed the theoretically predicted associations between mathematics-related emotions and their core antecedents and outcomes. For instance, students who reported higher levels of control (e.g., higher self-efficacy) and positive value (e.g., higher interest) also reported higher levels of positive emotions and lower levels of negative emotions. In turn, higher levels of positive emotions and lower levels of negative emotions were linked to higher motivation (e.g., effort), different learning styles (e.g., more self-regulation), and higher performance (i.e., better grades). This aligns well with previous research demonstrating the influence of control and value appraisals on mathematics-related emotions (e.g., Frenzel et al., 2007a, 2007b) and the effect of these emotions on performance (e.g., Pekrun et al., 2017). Besides these main findings, it is noteworthy that the relationships between achievement emotions and performance were substantial (e.g., the latent correlation between enjoyment and grades was *r* = 0.46). Across both studies, the AEQ-M scales demonstrated good reliability (i.e., it allows researchers to measure emotions with sufficient precision; Cronbach’s α ranging from 0.84 to 0.96) and were correlated with each other in meaningful ways (e.g., higher levels of one negative emotion were associated with higher levels of other negative emotions; 0.14 ≤*|r|*≤ 0.86).

Our findings are of great relevance for mathematics education for several reasons. Mathematics is a core subject in school curricula around the world and commonly accompanies students through their entire school life and beyond, especially in STEM-related occupational careers, but also more generally in understanding science and the world (e.g., statistics about diseases and health behavior related to the Covid-19 pandemic). Understanding the emotions students experience in mathematics is therefore of paramount importance (Schukajlow et al., 2017), not only because the emotional experiences of students in mathematics class should be studied as an outcome variable in itself, but also because emotions are an important predictor of mathematics achievement (Kim et al., 2014). A psychometrically sound, comprehensive, and valid instrument for assessing emotions in mathematics is therefore indispensable, with the need for such an instrument already demonstrated by existing research capitalizing on the AEQ-M. For instance, the AEQ-M has been used to examine the sources of gender differences in mathematics anxiety (Frenzel et al., 2007a, 2007b) and to investigate the effects of different special education support measures in mathematics on student’s emotions (Holm et al., 2020).

The extended AEQ-M scales developed in Study 2 will allow researchers to measure systematically the different components of achievement emotions (i.e., the affective, cognitive, motivational, and physiological/expressive processes of which emotions are composed). In future studies, it could thus be investigated whether these components are differentially affected by control and value appraisals and whether there are differences in the associations with performance. For instance, the cognitive component of mathematics anxiety (e.g., worries) might be more strongly affected by low levels of perceived control and more substantially associated with higher levels of performance than other components of anxiety (Roos et al., 2021a, 2021b; see also Barroso et al., 2021). This would have important practical implications for mathematics education, as it might guide the design of interventions (e.g., strengthening self-efficacy beliefs to reduce anxiety).

The extended AEQ-M scale may also inform psychometric research, as the results from studies may allow researchers to compare the components of the AEQ-M with the use of single-item measures to capture achievement emotions (Gogol et al., 2014). Moreover, the systematic coverage of emotion components in the extended AEQ-M scales permits an examination of the interplay of these components across different emotions (e.g., anxiety and boredom might share similar motivational processes such as the urge to leave a situation; Lange & Zickfeld, 2021). This would again greatly benefit mathematics education by identifying possible synergy effects among interventions.

In the present research, we developed extended AEQ-M scales to cover systematically all theoretically assumed components of emotions. However, the expanding of a scale may be a double-edged sword, as adding items to scales can be beneficial in terms of increasing reliability and ensuring that all relevant aspects of a construct may be captured, but it may also render the scale less convenient to administer (e.g., increasing time, decreasing compliance). This is particularly relevant for repeated assessments, for instance, in the context of experience sampling studies (Goetz et al., 2016). Experience sampling assesses emotions at the moment of their experience, which is increasingly used to study students’ and teachers’ emotions in the domain of mathematics (e.g., Bieg et al., 2017). A complementary approach would be the development of scales with fewer items that still cover each emotion component, which would address the balance between brevity and comprehensiveness. In terms of the domain-general AEQ, such a short-form has already been developed (the AEQ-S uses four items to measure all components of an emotion; Bieleke et al., 2021). It would therefore be possible to develop similar short-form versions of the AEQ-M, as adapting an already domain-specific questionnaire to a different domain may be less complex than adapting versions of a domain-specific questionnaire like the AEQ.

There are also some limitations of the present research that should be considered when interpreting our findings. Firstly, both studies are based on samples from German secondary schools. And while there already is evidence on the invariance of the AEQ-M across cultures (Frenzel et al., 2007a, 2007b), it would be desirable to investigate whether our results generalize to other age groups and educational settings (e.g., university students). This seems particularly important for the newly developed extended AEQ-M scales. Relatedly, we did not focus on gender differences as CVT assumes structural equivalence across gender—for instance, the association between control and value, and achievement emotions should be similar across female and male students (e.g., Pekrun et al., 2007; for empirical evidence, see Frenzel et al., 2007a, 2007b). Moreover, measurement equivalence of the AEQ-M across genders has already been demonstrated elsewhere (e.g., Moreira et al., 2019).

Secondly, our validation of the AEQ-M in terms of the core antecedents and outcomes of achievement emotions relied on self-reports. This mirrors the approach commonly taken in related research on achievement emotions (Bieleke et al., 2021; Pekrun et al., 2011), however it should still be complemented by more objective measures in future research. For example, physiological measures provide information beyond self-report and could be used to further validate the AEQ-M (Roos et al., 2021a, 2021b).

Thirdly, the fit of models representing different component structures of emotions did not always meet the thresholds recommended in the literature (e.g., for the learning- and test-related enjoyment scales). This may indicate a need for further refinement of these scales in future research, especially when the focus is on distinguishing the different components of emotions. However, it should be noted that these recommended cut-off criteria were derived from simulated datasets and are often not met with data sets derived from more complex studies, suggesting that they should be used with caution (Heene et al., 2011; Marsh et al., 2004). Relatedly, future research might use different analytic approaches to investigate the assumptions behind the AEQ-M. For instance, the context-dependency of mathematics-related emotions and their component structure could be jointly examined in one comprehensive model rather than in two separate steps. This added complexity might allow the examination of more fine-grained hypotheses (e.g., whether context-dependency holds across the different components of emotions).

Fourthly, the research design in Study 1 is correlational and does not allow us to draw causal inferences about the relations between achievement emotions and their antecedents and outcomes, or to capture mediated relations between these constructs. While the observed correlations are in line with CVT propositions, experimental or longitudinal data are necessary to examine the causal effects generating these correlations (see, e.g., Forsblom et al., 2022; Pekrun et al., 2017).

## Conclusion

Across two independent studies, we examined the internal structure and external relations of mathematics-related emotions measured with the AEQ-M, a widely used instrument that has not been published yet and that lacks dedicated evaluations of the validity of its assumptions. Our results indicate that the structural properties of the AEQ-M correspond closely to predictions that can be derived from the control-value theory of achievement emotions, and these results are similar to those observed for achievement emotions in other school domains. Specifically, mathematics-related emotions depend on the academic context in which they occur (i.e., class, learning, and test), represent a set of interrelated psychological processes (i.e., affective, cognitive, motivational, and physiological/expressive components), and are linked to their assumed antecedents (control, value) and outcomes (motivation, learning strategy, achievement). We introduced a set of extended AEQ-M scales that researchers in mathematics education can use to conduct a valid, reliable, and systematic examination of the component structure of several mathematics-related emotions in future studies.

## Appendix 1. AEQ-M Instructions

Note: Differences between contexts are provided in square brackets: [class | learning | test]

“[Attending classes | Studying and doing homework assignments | Tests and exams] in mathematics can induce different feelings. This part of the questionnaire refers to emotions you may experience when [being in math classes | studying and doing homework in math | taking tests or exams in math]. Before answering the following questions, please recall some typical situations of [being in a math class | studying and doing assignments in math | taking tests or exams in math] which you have experienced.”

1. Before [class | studying | taking the test/exam]: “The following questions pertain to feelings you may experience BEFORE [attending math classes | studying and doing homework in mathematics | taking a test or an exam in mathematics]. Please indicate how you feel, typically, before [you go to a math class | you begin to study or do an assignment in math | taking a test or an exam in math].”
2. During [class | studying | taking the test/exam]: “The following questions pertain to feelings you may experience DURING [math classes | studying and doing homework in mathematics | taking a test or an exam in mathematics]. Please indicate how you feel, typically, during [math classes | studying and doing homework in math | taking a test or an exam in math].”
3. After [class | studying | taking the test/exam]: “The following questions pertain to feelings you may experience AFTER [having attended a math class | having studied or done homework in mathematics | taking a test or an exam in mathematics]. Please indicate how you feel, typically, after [having attended a math class | having studied or done homework in math | taking a test or an exam in math].”

## Appendix 2. AEQ-M Scales

Note: Italicized items belong to the extended AEQ-M scales

**Enjoyment**

-  I look forward to my math class
-  I enjoy my math class
-  The material we deal with in mathematics is so exciting that I really enjoy my class
-  I enjoy my class so much that I am strongly motivated to participate
- *I am cheerful in my math class*
- *I look forward to learning a lot in my math class*
- *I am happy that I understood the material*
- *I could listen enthusiastically for hours*
- *In my math class, I feel my heart pounding with joy*
- *I smile at my teacher with joy about my math class*
-  When doing my math homework, I am in a good mood
-  I am happy that I understand the material
-  I enjoy doing my math homework so much that I am motivated to do extra assignments
- *In math, I look forward to doing the homework*
- *I enjoy the homework in math*
- *I am happy that the assignments are so exciting*
- *I am happy that the math homework did not cause me any problems*
- *Because I enjoy math, I engage in it more than I need to*
- *When it goes well, I feel my heart pounding with joy*
- *When math homework went well, I beam with joy*
-  I enjoy taking tests in mathematics
-  Because I look forward to getting a good grade, I study hard for the test
-  I think that things are going great
- *Math tests are tests that I enjoy*
- *I am happy that I can show what I have learned right away*
- *I think I did pleasingly well on the math test*
- *When I notice that I am doing well, I try harder*
- *When the math test goes well, I smile with joy*
- *I am so happy that I feel warm*
- *I feel my heart beat faster with joy*

**Pride**

-  I think I can be proud of my knowledge in mathematics
-  I am proud of my contributions to the math class
-  After having done my math homework, I am proud of myself
-  I am very motivated because I want to be proud of my achievements in mathematics
-  After a math test, I am proud of myself
-  I am proud of how well I have done on the math test

**Anger**

-  I am annoyed during my math class
-  I am so angry during my math class that I would like to leave
-  I get angry because the material in mathematics is so difficult
-  I get irritated by my math class
- *I am upset*
- *My math class really makes me angry*
- *I am irritated with the math teacher*
- *I would like to take my anger out on my classmates*
- *I am getting hot with anger*
- *I am boiling with rage inside*
- *In my math class, I look irritated*
-  My mathematics homework makes me angry
-  I get angry because my math homework occupies so much of my time
-  I am so angry that I would like to throw my homework into the thrash
- *I get angry when I do math homework*
- *I get angry that I have to do so much homework in math*
- *I would prefer not to start at all out of anger about the math homework*
- *The math homework makes me so angry that I would like to stop doing it*
- *Even before I do the math homework, I get upset with anger*
- *When I have to sit and do math homework for a long time, I get all restless with anger*
- *When I have done math homework for a long time, I get all tense with anger*
-  I am so angry that I would like to tear the exam paper into pieces
-  I am annoyed that the teacher asks such difficult questions
- *I am angry*
- *After the math test, I feel a real anger in my stomach*
- *After a test, I'm angry*
- *I feel angry about how the math test went*
- *I wish my math teacher would get lost*
- *I notice how my fists clench in anger during the math test*
- *I feel my blood rush to my head with anger*
- *I'm so angry that I feel hot all over*

**Anxiety**

-  When thinking about my mathematics class, I get nervous
-  I worry if the material is much too difficult for me
-  When thinking of my math class, I get queasy
-  Math scares me so much that I would rather not attend school
- *I am afraid of my math class*
- *I am nervous in my math class*
- *Even before my math class, I worry if I will understand enough*
- *I worry if I will understand less than the others*
- *Because I'm afraid I won't be able to follow the math class, I try extra hard*
- *Because I am afraid of saying something wrong, I would prefer not to speak at all*
- *I am very anxious because of my fear*
- *In my math class, I start to sweat from fear*
-  I worry whether I will ever be able to completely understand the material
-  I start sweating because I am worried I cannot complete my assignments in time
-  I am tense and nervous
-  I’m so scared of my math assignments that I would rather not start them
- *I am afraid of math assignments*
- *When I get stuck on my math assignments, I get scared*
- *I worry that my math assignments will be too difficult for me*
- *I worry if I will ever get it right*
- *Because I am afraid that I won't be able to do my math assignments, I try even harder*
- *When I do my math assignments, I have to get a funny feeling in my stomach from nervousness*
- *When I can't complete the math assignments, my heart pounds from anxiety*
-  When taking the math test, I am tense and nervous
-  When taking the math test, I worry I will get a bad grade
-  I am very nervous
-  Even before I take the math test I worry I could fail
-  I am so anxious that I would rather not take the math test
-  When I have an upcoming math test, I get sick to my stomach
-  I am so anxious that I can’t fully concentrate
- *I worry that the tasks are too hard for me*
- *I am so scared that I wish I was far away*
- *During a math test, my hands get clammy*
- *When I am quizzed, I have wobbly knees*
- *I am so nervous that I can't remember what I've learned*

**Shame**

-  When I say something in my math class, I can tell that my face gets red
-  I am ashamed that I cannot answer my math teacher’s questions well
-  When I say something in my math class, I feel like embarrassing myself
-  I am embarrassed about my lack of knowledge in mathematics
-  When I don’t understand something in my math homework, I don’t want to tell anybody
-  When I discuss the homework assignments with my classmates, I avoid eye contact
-  After taking a test in mathematics, I feel ashamed
-  I start sweating because my performance on the math exam embarrasses me

**Hopelessness**

-  I feel down
-  During the math test, I feel hopeless
-  I keep thinking that I don’t understand the material
-  I keep thinking that I will never get good grades in mathematics
-  I would prefer to give up
-  I have no energy
- *Because I feel so hopeless, I don't make any more effort*
- *I feel paralyzed*
- *I almost have to cry because I don't know what to do*

**Boredom**

-  I think the mathematics class is boring
-  I can’t concentrate because I am so bored
-  I am so bored that I can’t stay awake
- *I am bored*
- *In my math class, my mind is often somewhere else*
- *Being bored, I think that I don't really care about the material*
- *When I am bored, I think to myself: If only the math class were already over!*
- *In my math class I daydream*
- *I constantly look at the clock, because the time does not pass*
- *I try to distract myself out of boredom*
- *I get restless because I am just waiting for the math class to finally be over*
- *I notice how I sink down in my chair from boredom*
-  Just thinking of my math homework assignments makes me feel bored
-  My math homework bores me to death
-  I’m so bored that I don’t feel like studying any more
- *I'm thinking about how boring the math homework is again today*
- *My mind is somewhere else entirely*
- *I am bored thinking to myself that there is not much point in doing these assignments*
- *The assignments are so boring that I don't feel like starting*
- *Out of boredom, I would rather do something I enjoy*
- *When doing math homework, I get tired quickly from boredom*
- *I have to yawn because of boredom*
